# Reciprocal interplay between asporin and decorin: Implications in gastric cancer prognosis

**DOI:** 10.1371/journal.pone.0255915

**Published:** 2021-08-11

**Authors:** Dipjit Basak, Zarqua Jamal, Arnab Ghosh, Pronoy Kanti Mondal, Priyanka Dey Talukdar, Semanti Ghosh, Biswadeep Ghosh Roy, Ranajoy Ghosh, Aniket Halder, Abhijit Chowdhury, Gopal Krishna Dhali, Bitan Kumar Chattopadhyay, Makhan Lal Saha, Abhimanyu Basu, Sukanta Roy, Chitranjan Mukherjee, Nidhan Kumar Biswas, Urmi Chatterji, Shalini Datta

**Affiliations:** 1 Human Genetics Unit, Indian Statistical Institute, Kolkata, India; 2 Cancer Research Lab, Department of Zoology, University of Calcutta, Kolkata, India; 3 National Institute of Biomedical Genomics, Kalyani, India; 4 Crystallography and Molecular Biology Division, Saha Institute of Nuclear Physics, Kolkata, India; 5 The School of Digestive and Liver Diseases, Institute of Post Graduate Medical Education and Research, Kolkata, India; 6 Department of Surgery, Institute of Post Graduate Medical Education and Research, Kolkata, India; 7 Delhi Technological University, Delhi, India; University of Crete, GREECE

## Abstract

Effective patient prognosis necessitates identification of novel tumor promoting drivers of gastric cancer (GC) which contribute to worsened conditions by analysing TCGA-gastric adenocarcinoma dataset. Small leucine-rich proteoglycans, asporin (*ASPN*) and decorin (*DCN*), play overlapping roles in development and diseases; however, the mechanisms underlying their interplay remain elusive. Here, we investigated the complex interplay of asporin, decorin and their interaction with TGFβ in GC tumor and corresponding normal tissues. The mRNA levels, protein expressions and cellular localizations of ASPN and DCN were analyzed using real-time PCR, western blot and immunohistochemistry, respectively. The protein-protein interaction was predicted by in-silico interaction analysis and validated by co-immunoprecipitation assay. The correlations between ASPN and EMT proteins, VEGF and collagen were achieved using western blot analysis. A significant increase in expression of ASPN in tumor tissue vs. normal tissue was observed in both TCGA and our patient cohort. DCN, an effective inhibitor of the TGFβ pathway, was negatively correlated with stages of GC. Co-immunoprecipitation demonstrated that DCN binds with TGFβ, in normal gastric epithelium, whereas in GC, ASPN preferentially binds TGFβ. Possible activation of the canonical TGFβ pathway by phosphorylation of SMAD2 in tumor tissues suggests its role as an intracellular tumor promoter. Furthermore, tissues expressing ASPN showed unregulated EMT signalling. Our study uncovers ASPN as a GC-promoting gene and DCN as tumor suppressor, suggesting that ASPN can act as a prognostic marker in GC. For the first time, we describe the physical interaction of TGFβ with ASPN in GC and DCN with TGFβ in GC and normal gastric epithelium respectively. This study suggests that prevention of ASPN-TGFβ interaction or overexpression of DCN could serve as promising therapeutic strategies for GC patients.

## Introduction

According to GLOBOCAN 2018, about 782,685 people have died from gastric cancer (GC) and almost a million new cases have been added since then [1]. GC is second most common cause of cancer-related deaths in young Indian patients below 45 years [2]. The five-year survival rate of GC is unmitigable, despite great advances in surgery and adjuvant treatments [3–5]. Therefore, there is a dire need for the identification and characterization of novel genetic biomarkers impacting prognosis for better management of GC.

The Cancer Genome Atlas (TCGA) research network has revealed molecular classification of GC, shedding light on the previously unexplored realms of GC [6, 7]. RNA-Seq data from TCGA on stomach adenocarcinoma cohort (STAD) was analyzed to classify genes, which positively or negatively correlated with the stages of GC. Several genes were identified and pathway analysis performed. One of the most interesting candidates that emerged from the pathway analysis was asporin (ASPN), a small leucine-rich proteoglycan (SLRP), which is a component of extracellular matrix organization protein [8–10]. Previous studies focused its roles in orthopedic diseases [11–14]; however, recent findings have reported it to have an oncogenic role in several cancers—pancreatic, colorectal, gastric, prostate cancers, and in invasive ducal breast carcinoma [15–21]. Interestingly, in triple-negative breast cancer, it is reported to function as a tumor suppressor gene [22]. Therefore, targeting the specific molecular signaling pathway of ASPN may unravel its role in precision medicine. In addition, another SLRP which warranted concomitant attention was decorin (DCN), a putative tumor suppressor gene [23–25].

The involvement of transforming growth factor beta (TGFβ) in GC progression has been unequivocally studied, and its interaction with SLRPs has also been established [23–32]. The interaction between ASPN and TGFβ has been observed in the pathogenesis of osteoarthritis [30, 33–35], though its precise molecular interaction in GC progression remains elusive and necessitates further investigation. However, in the context of cancer progression, TGFβ was the first growth factor with which DCN was identified to interact [28, 31, 36, 37]. It has experimentally been proven that DCN effectively inhibits TGFβ-induced cancer progression and proliferation in different cancer cell lines [38, 39]. In vivo tumor treatment studies with a recombinant DCN core protein have thereby been carried out with considerable success [40, 41].

The relationship between ASPN and DCN, along with their specific interactions with TGFβ, in regulating the progression of GC has not been investigated yet. To resolve this lacuna, we data-mined the TCGA-STAD database and employed our sample library to validate the results. The relation of ASPN and DCN gene expression with patient survival was determined by TCGA and other GEO datasets on GC. Molecular interaction of the SLRPs with TGFβ was determined. Involvement of TGFβ in the regulation of EMT phenotype, collagen synthesis and angiogenesis were assessed in aggressive tumor tissues with high ASPN expression and low DCN expression. This study reveals the possible role of ASPN and DCN in tumor progression and provides evidence that both ASPN and DCN can serve as novel prognostic biomarkers of GC. Since single prognostic biomarkers can often be inconclusive and ambiguous, dual markers with antagonistic roles could provide a more objective utility for the prognosis of GC.

## Materials and methods

### Ethical approval and collection of tissue samples from gastric adenocarcinoma patients

All participants gave their written informed consent, and the study was approved by the ethics committee of the Institute of Post Graduate Medical Education and Research (I.P.G.M.E. & R.) and S.S.K.M. Hospital, Kolkata, India and Indian Statistical Institute (I.S.I), Kolkata. The cohort of patients undergoing surgery have a distinctive pathologic and clinical diagnosis of gastric adenocarcinoma and designated as OT cohort in our study. The OT cohort patients who had undergone curative total or subtotal gastrostomy at the Institute of Post Graduate Medical Education and Research (I.P.G.M.E. & R.), Kolkata between 2017 and 2019 did not receive any chemotherapy or radiotherapy prior to surgery. Gastric adenocarcinoma tissues and their corresponding normal tissues from same patient were collected from 42 patients in the OT cohort (Table 1). Patients with a history of other primary cancers and patients positive for Hepatitis B, Hepatitis C, and HIV were excluded from this study. The patients, who had undergone pre-operative endoscopic biopsy at the same institute between 2017 and 2019, are designated as endoscopic biopsy cohort (ENDO cohort) in our study. Gastric adenocarcinoma biopsy tissues and their corresponding normal biopsy tissues were collected from stomach tumor growth of the 35 patients in the ENDO cohort (S3 Table). They have a distinctive pathologic and clinical diagnosis of gastric adenocarcinoma and did not receive any chemotherapy or radiotherapy prior to endoscopy. All collected tissue samples were stored in RNA*later*^TM^ (Invitrogen, Thermo Fischer Scientific, USA) solution until DNA and RNA isolation. Following Lauren’s classification, collected GC tissue samples were classified into two main types: Intestinal and Diffuse type and were cross-verified by two pathologists.

**Table 1:** Demographic table of OT cohort patients.

Characteristics	Number of OT cohort patients (%)	Overexpressed in tumor	Under expressed in tumor	Fischer’s test p value
**Gender**				1
Male	31/42(~74%)	19	12	
Female	11/42(~26%)	7	4	
**Age (years)**				1
>60	12/42(~29%)	7	5	
≤60	30/42(~71%)	19	11	
**Lauren’s subtype**				1
Intestinal	18/42(~43%)	11	7	
Diffuse, diffuse with signet ring	24/42(~57%)	15	9	
**Differentiation**				0.352
Poor, Moderate	24/42(~57%)	24	13	
Well	18/42(~43%)	2	3	
**Tumor stage**				**0.047** *
I + II	13/42(~30%)	5	8	
III + IV	29/42(~70%)	21	8	
			
**Lymph Node Metastasis**				**0.008** **
N0, N1	33/42(~79%)	17	16	
N2, N3	08/42(~21%)	9	0	
**Distant Metastasis**				0.067
Negative	36/42(~86%)	20	16	
Positive	06/42(~14%)	6	0	

Bold values indicate a significant difference.

Note:

* p < 0.05

** p < 0.01: p-values determined with Fischer’s exact test.

### Stage-wise identification of differentially expressed genes in stomach adenocarcinoma from publicly available databases

To detect differentially expressed genes in stomach adenocarcinoma (STAD), we used mRNA expression data of patient samples from TCGA. RNAseq count data of 60483 genes were obtained from Genomics Data Commons (GDC) portal (https://portal.gdc.cancer.gov/) for STAD cancer type (n = 375) and normal tissue samples (n = 32). Information about histopathological stages of cancer samples were obtained from cBioPortal (https://www.cbioportal.org/). All non-coding genes and protein coding genes that are expressed at a very low level and in less than 50% of cases or controls were filtered out for downstream analysis. Data on STAD patients with incomplete histopathological staging information were removed. Differential gene expression (DGE) analysis was performed using the DESeq2 method using high-throughput sequence (HTSeq) count data obtained from GDC portal. Significantly differentially expressed genes (|log2 fold change| > = 1 and FDR corrected p < 0.1) were detected separately for following comparisons: i) stage 1 and 2 tumor (n = 98) vs normal (n = 32) as controls, ii) stage 3 tumor (n = 168) vs normal (n = 32) and iii) stage 4 tumor (n = 100) vs normal (n = 32). DGE analysis detected 1183, 1254, and 1115 genes to be up regulated in stages 1–2, stage 3, and stage 4 groups of STAD patients respectively. Out of these, 58 genes showed a stage-wise increasing trend in expression fold change (S5 Table). Pathway enrichment analysis was performed with these 58 genes using the Reactome webserver (https://reactome.org/).

### RNA isolation and gene expression quantification by RT-PCR

Total RNA from the gastric tumor and adjacent normal tissues (control tissue from the same patient but from normal site) from both cohorts were extracted using miRNeasy kit (Cat#217004, Qiagen, Denmark) following manufacturer’s instructions. RNA quality (A260/A280 ratio) and quantification were determined using Nanodrop^TM^ 2000 (Thermo Scientific, Wilmington, Delaware, USA) spectrophotometer. About 2 μg of RNA was reverse transcribed using the PrimeScript^TM^ first strand cDNA Synthesis Kit (Cat# 6110; Takara Bio Inc., Shiga, Japan) at 50°C for 60 min, followed by 70°C for 15 min. Quantitative real-time PCR was performed using iTaq Universal SYBR Green Supermix (Bio-Rad, Hercules, California, USA) on a 7900HT Fast Real-time PCR system (Applied Biosystems, Foster City, CA, USA). Primers were designed to target specific genes for Real-time PCR (RT-PCR) as mentioned in S1 Table. During all preliminary studies, we have checked the melting curves for our RT-PCR experiments to evaluate the specificity of the all the primers we had designed. To quantify gene expression through RT-PCR were performed using human beta actin (ACTB) as standard reference [42]. Finally, the mRNA expression level was estimated using the standard 2^-ΔΔCt^ method for each gene [42, 43].

### Immunohistochemical staining

Immunohistochemical staining (IHC) of ASPN was performed on a subset of 12 GC patients of OT cohort. IHC was performed using SCYTEK CRFTM Anti-Polyvalent HRP Polymer (DAB) Stain kit (SCYTEK Laboratories Inc., Logan, Utah, USA) according to the manufacturer’s protocol. The tumor and adjacent normal tissue sections were deparaffinized and hydrated. Antigen was retrieved using Tris-EDTA buffer (pH 9.0). Non-specific peroxidase activity was achieved with peroxide blocking chemical provided in the kit, followed by serum-free protein blocking agent for 10 min (SCYTEK Laboratories Inc., Logan, Utah, USA. Immunostaining of ASPN was performed for 90 min using anti-ASPN antibody (Cat# ab58741, Abcam, Cambridge, MA, USA), diluted to 1:250. CRF anti-polyvalent HRP was used as the secondary antibody and chromogenic detection was carried out using DAB chromogen (1:20) (SCYTEK Laboratories Inc., Logan, Utah, USA). Finally, immunostained slides were counterstained with hematoxylin. Using the semi-quantitative immunoreactivity scoring (IRS), IHC staining was scored independently by two pathologists who were unaware of the patients’ clinical data. Images of IHC stained slides were captured at 10X and 40X magnification. The IHC staining was scored by a semi-quantitative method according to the percentage and intensity of positively stained cells. The four scoring categories were “0” (negative staining); “1” (weak staining); “2” (moderate staining); and “3” (strong staining).

### Bioinformatics prediction and modelling of protein-protein interaction

To investigate protein-protein interactions, three dimensional models were needed for protein of interest. Therefore, amino acid sequences of the proteins were searched in BLASTp against Protein Data Bank Database to find a suitable template for homology modeling (Supplementary methods in S1 File).

### Co-immunoprecipitation

Gastric tumor and adjacent normal tissue from OT cohort were washed in ice-cold phosphate buffer saline (PBS) and protein extracted by homogenizing tissues in radioimmunoprecipitation assay (RIPA) buffer followed by centrifugation at 12000 rpm for 15 mins at 4°C. Protein lysate in the supernatant was collected. Whole tissue lysates (3 pairs) were incubated with anti-TGF-β1 antibody (Cat# MAB1835, R & D Systems, Minneapolis, USA) (1:1000) along with 50 μl of agarose-protein A/G beads 4°C overnight to form immunocomplexes. After washing with lysis buffer, 100 μg total proteins were resolved by 10% SDS-PAGE and the immunocomplexes were analyzed by immunoblotting with anti-DCN (Cat# AF143, R & D Systems, Minneapolis, USA) (1:1000) and anti-ASPN (Cat# ab58741, Abcam, Cambridge, MA, USA) (1:1000) antibodies. After incubation of these immunocomplexes with peroxidase-conjugated secondary antibodies (1:3000) for 2 hours at room temperature, the amount of TGFβ that co-immunoprecipitated with DCN and ASPN was documented using the Gel Doc XR type imaging system. The band intensities were quantified using Image J software.

### Western blot

Three tumor-normal pairs of patient tissue lysates of OT cohort were used to perform most of the western blots using appropriate antibodies. Gastric tumor and adjacent normal tissue were washed in ice-cold PBS. Protein was extracted by homogenizing tissues in RIPA buffer followed by centrifugation at 12000 rpm for 15 min at 4°C and protein lysate in the supernatant was collected. Total protein was quantified by the Bradford assay. About 30 μg of total protein was electrophoresed and incubated with 5% non-fat skim (or dry, since the protein contents vary) milk buffer with primary antibodies. The primary antibodies (1:1000 dilution) used in this study were anti-ASPN (Cat# ab58741, Abcam, Cambridge, MA, USA), anti-DCN (Cat# AF143, R & D Systems, Minneapolis, USA), anti-E-cadherin (Cat# AF748, R & D Systems, Minneapolis, USA), anti-N-cadherin (Cat# AF6426, R & D Systems, Minneapolis, USA), anti-Fibronectin (Cat# ab2413, Abcam, Cambridge, MA, USA), anti-TGFβ1 (Cat# MAB1835, R & D Systems, Minneapolis, USA), anti-SMAD2 (Cat#D43B4, Cell Signaling Technology, Massachusetts, USA), anti-pSMAD2 (Cat# 138B4, Cell Signaling Technology, Massachusetts, USA), anti-Col1A1 (Cat# sc-80760, Santa Cruz Biotechnology, Texas, USA), anti-VEGF (Cat# sc-7269, Santa Cruz Biotechnology, Texas, USA), anti-ACTB (Cat# sc-47778, Santa Cruz Biotechnology, Texas, USA) at 4°C overnight. Membranes were then washed and incubated with peroxidase-conjugated secondary antibodies (1:3000) for 2 hours at room temperature. Proteins were visualized using enhanced chemiluminescence (ECL, Bio-RAD, USA) according to the manufacturer’s instructions, followed by quantification of bands using Image J software.

### Protein-protein interaction (PPI) network construction

The functional interactions between encoded proteins of studied genes were explored using the Search Tool for the Retrieval of Interacting Genes (STRING) database (http://string-db.org) [44]. STRING is a database of known and predicted protein-protein interactions. Interactions in STRING are derived from five main sources as Genomic Context Predictions, High-throughput Lab Experiments, (Conserved) Co-Expression, Automated Text mining and Previous Knowledge in Databases [45]. The PPI network was used to identify main interacting partners of ASPN and DCN.

### Survival analysis

We have categorised TCGA-STAD tumor into two groups based on gene expression level: a) low [gene expression < 25^th^ percentile of *ASPN* expression in normal tissue] and b) high [gene expression > = median + 2x standard-deviation *ASPN* expression in normal tissue]. Using survival R-package (https://github.com/therneau/survival) we have tested and rejected our null hypothesis (H_0_) that there is no difference in survival between low and high ASPN expressing groups of patients with the log-rank test. Kaplan-Meier (K-M) survival curve analysis was performed with the data from three publicly available gastric cancer Gene Expression Omnibus (GEO) datasets, namely, GSE62254, GSE15459, and GSE14210. For GEO data sets, the prognostic value of mRNA expression of aspirin in GC was analysed by using Kaplan-Meier plotter (http://kmplot.com/analysis/) [46]. In Kaplan-Meier plotter, cancer patients were divided into high and low expression group based on median values of mRNA expression and validated by K-M survival curves. Information about the number-at-risk cases, median values of mRNA expression levels, HRs, 95% CIs and *p*-values can be found at the K-M plotter webpage. Statically significant difference was considered when a *p* value < 0.05.

### Statistical analyses

All assays were repeated at least three times and the results represented the average. RT-qPCR was analyzed using a paired two-tailed *t*-test and clinico-pathological parameter studies were analyzed using Fisher’s exact test. All values of western blot were normalized against loading control. Differences between groups in western blot were evaluated using Student’s *t*-test data are presented as mean ± SD of at least three biological replicates as indicated in figure legends. *P* value <0.05 was considered statistically significant, and all statistical tests were two-sided.

## Results

### ASPN over expression predicts poor prognosis

**Fig 1:**
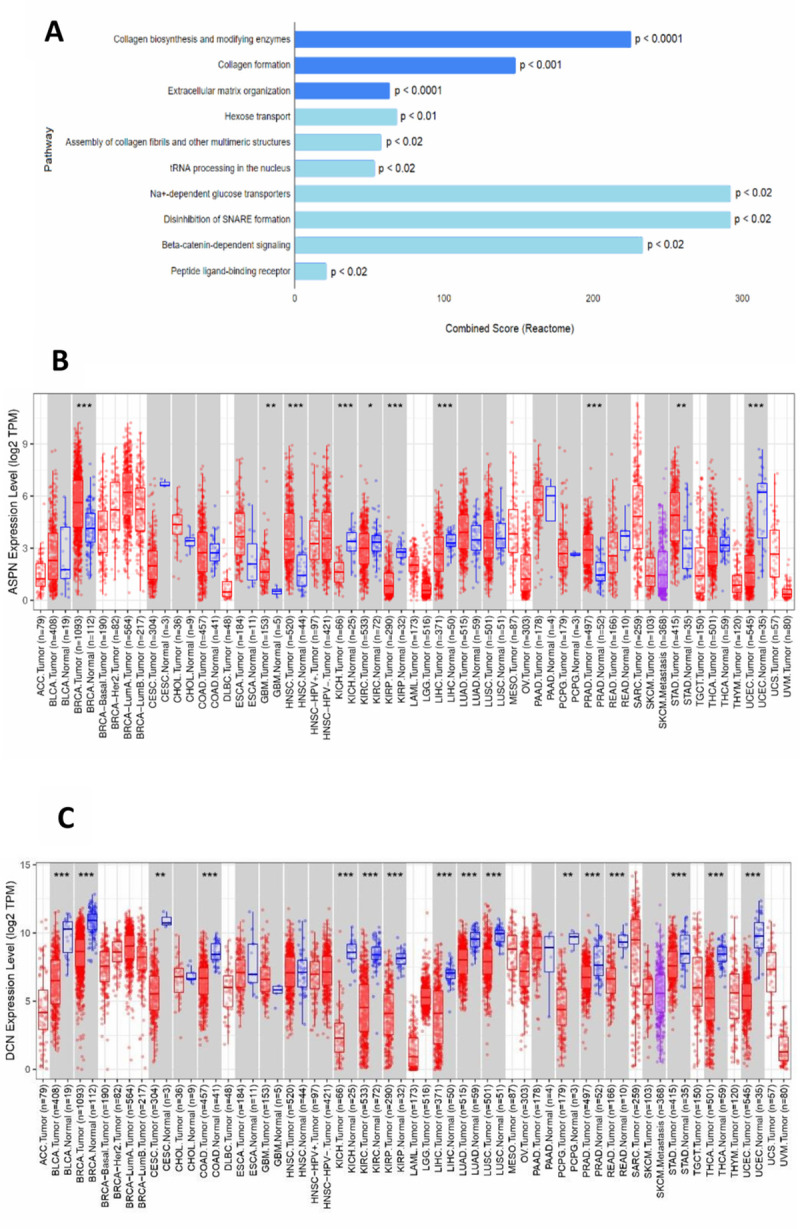
Transcriptomic data analysis of TCGA-STAD cohort. (A) Reactome Analysis of genes that showed stage-wise upregulation in GC on TCGA-STAD data. (B, C) The differential expression between tumor and adjacent normal tissues for ASPN (B) and DCN (C) across all TCGA tumors. Distributions of gene expression levels are displayed using box plots. The statistical significance computed by differential analysis (edgeR) on RNA-Seq raw counts is annotated by *p ≤ 0.05, **p ≤ 0.01, ***p ≤ 0.001. Up-regulated or down-regulated in the tumors compared to normal tissues for each cancer type are displayed in grey columns when normal data are available.

In order to identify prognostic markers of stomach adenocarcinoma we have identified transcriptomic signatures that are consistently modulated according to the stages of tumor progression. From publicly available TCGA stomach adenocarcinoma (STAD) datasets, we have downloaded RNA sequence data for 375 STAD tumors comprising of patients from 4 major pathological stages (Stage 1 = 32, Stage 2 = 98, Stage 3 = 168, Stage 4 = 100) and 32 adjacent- normal samples. Differential gene expression (DGE) analysis revealed sets of genes that are significantly deregulated in various stages as compared to adjacent normal. To identify prognostic signatures, we have focused on genes that showed a consistent up-regulation in a stage-wise manner. We found a total of 58 genes that harboured an incremental positive fold change with increasing pathological stages of the tumor as compared to adjacent normal tissue. Pathway enrichment analysis using these 58 stage-wise up regulated genes at Reactome revealed collagen synthesis and extracellular matrix organization pathways to be altered most significantly (p.adj <0.001). These pathways consist of *COL22A1*, *ITGA11*, *COL10A1*, *COL8A1*, *COLGALT1* and *ASPN* genes (Fig 1A). Pan cancer ASPN, DCN gene expression was assessed using TCGA data (Fig 1B and 1C).

**Fig 2:**
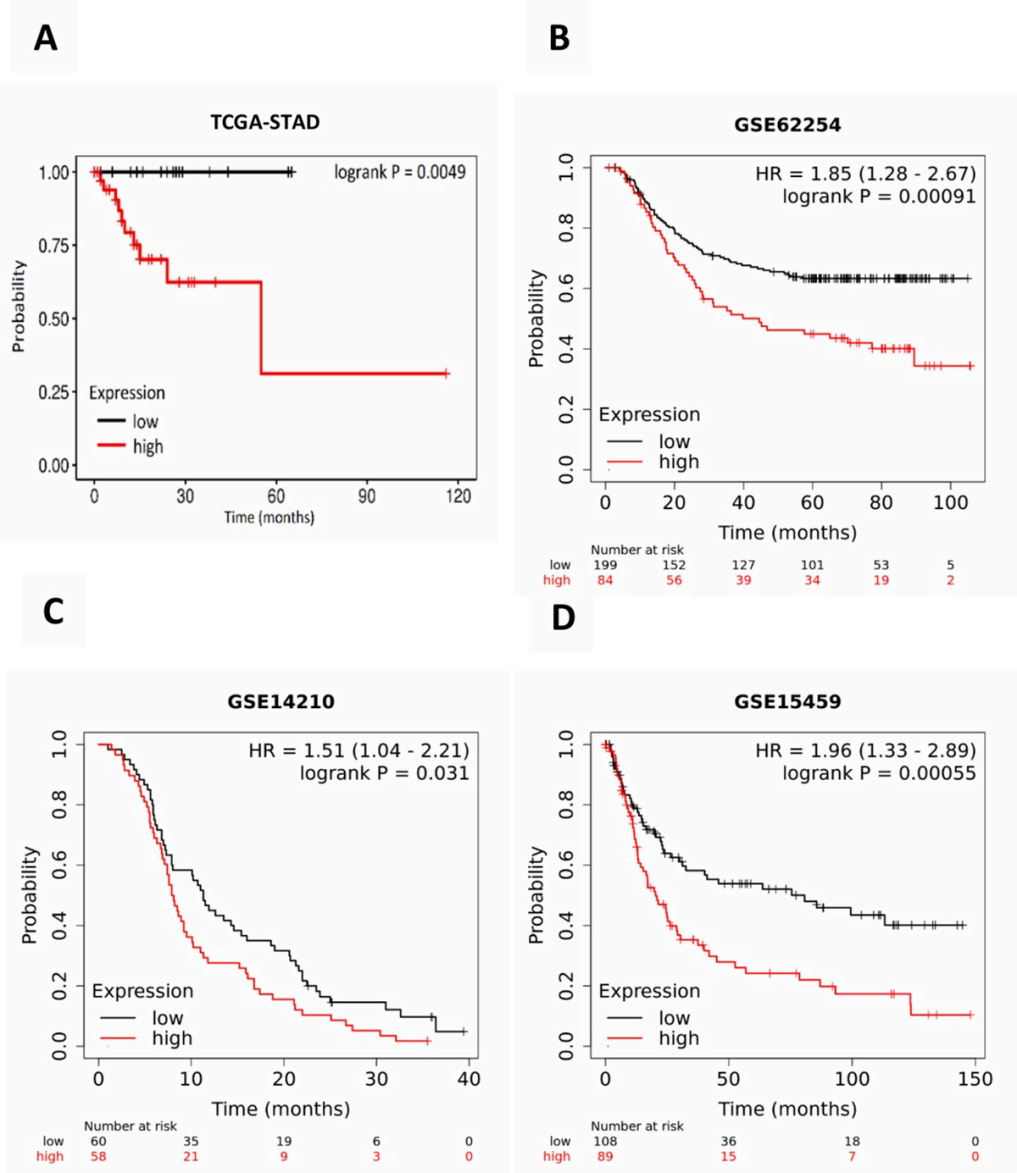
Kaplan-Meier plots of the survival analysis of GC patient data: Kaplan-Meier plots of the survival analysis of GC patient data from the TCGA RNA sequencing datasets (A) and three publicly available Gene Expression Omnibus (GEO) datasets, namely, (B) GSE62254 ACRG (Asian Cancer Research Group, N = 300) gastric cohort, (C) GSE14210 (metastatic gastric cancer patients, N = 145) and (D) GSE15459 (primary gastric tumors from Singapore patient cohort, N = 200) The patients are stratified in two groups (high expression in red and low expression in black) according to the expression profiles of ASPN. P-values for the significance of the difference between high and low expression were calculated using the log-rank test.

*ASPN* mRNA expression and prognosis of patients with gastric carcinoma was analysed based on TCGA and GEO datasets. Kaplan–Meier plot analysis showed that over expression of *ASPN* was associated with worse overall survival in the TCGA-STAD patient cohort (log-rank test *p* = 0.0049) (Fig 2A). To validate the prognostic value of *ASPN*, we obtained the *ASPN* expression data from three independent GEO datasets of GC, GSE62254 (Fig 2B) GSE15459 (Fig 2C) GSE14210 (Fig 2D). Kaplan–Meier plot showed that augmented expression of *ASPN* is associated with worse overall survival in all four datasets.

### Expression of *ASPN* and *DCN* in GC study cohort

**Fig 3:**
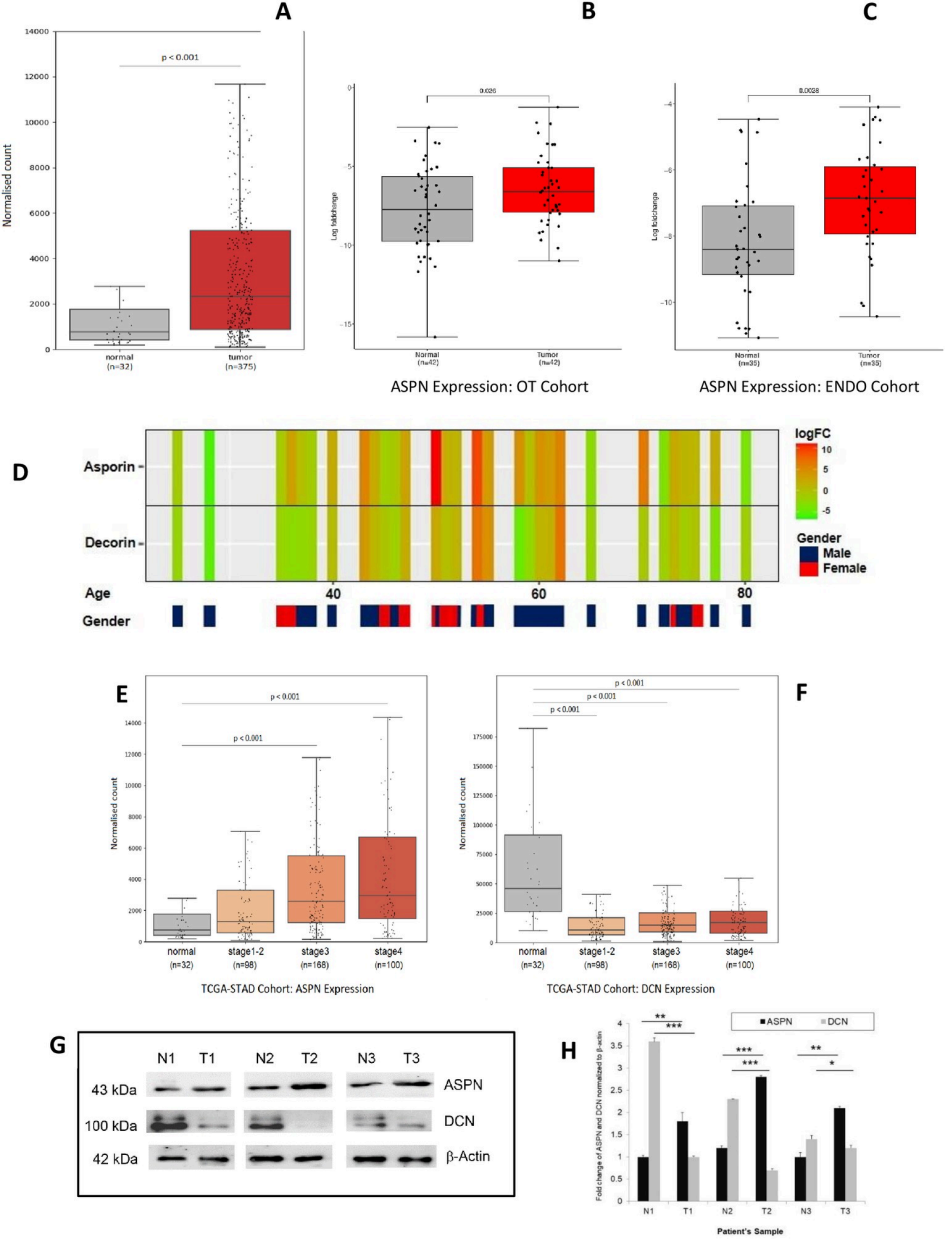
*ASPN* is upregulated in gastric tumors. (A) *ASPN* is upregulated in TCGA RNA Seq dataset (n = 407, mean ± SD, ***p ≤ 0.00,1 likelihood ratio test using DESeq2 method); (B, C) Gene expression analysis using quantitative real-time PCR suggests *ASPN* is highly expressed in gastric tumor tissue compare to adjacent normal in approx. 61.9% and 61.53% patients in OT Cohort and ENDO Cohort respectively. (n = 42 for OT Cohort and n = 35 for ENDO Cohort, mean ± SD, *p ≤ 0.05, **p ≤ 0.01, ***p ≤ 0.001, paired two tailed *t* test). (D) Heat map of 42 samples based on *ASPN* and *DCN* gene expression in our OT cohort. (E, F) *ASPN* and *DCN* expression was statistically associated with tumor stage: ASPN is overexpressing with increasing cancer stage whereas DCN showed an opposite result. (n = 398, mean ± SD, *p ≤ 0.05, **p ≤ 0.01, ***p ≤ 0.001, likelihood ratio test using DESeq2 method) (G, H) Expression of *ASPN and DCN* in Gastric tissues, adjacent normal and tumor samples, analysed using western blot assay: T1, T2, T3 are tumor tissues and N1, N2, and N3 are respective corresponding normal tissues.Data were quantified, normalized with β-actin and plotted (n = 3, mean ± SD, *p ≤ 0.05, **p ≤ 0.01, ***p ≤ 0.001, student’s *t* test).

The demographic and clinico-pathological characteristics of the GC patient cohort are summarized in Table 1. Bar plots were used to compare the gene expression profiles across all 375 tumor samples and paired normal tissues from the TCGA database (Fig 3A). *ASPN* expression was confirmed to be up regulated in the majority of tumor samples (Fig 3D). We used relative quantitation by RT-PCR for evaluating the mRNA expression of *ASPN* and *DCN* in ENDO cohort from GC patients and validated the results in our OT cohort. Beta actin (*ACTB)* served as the endogenous control. *ASPN* gene expression was up regulated in 20 out of 35 patients (57.14%) in the endoscopic biopsy (ENDO) cohort (Fig 3C). In OT cohort tumor samples, we found up regulation of *ASPN* in 26 out of 42 samples (61.9%; Fig 3B), while *DCN* was down regulated in 22 out of 42 samples (52.4%; S1 Fig).

*DCN*, a well-studied SLRP was predominantly down regulated in tumor samples. The association between *ASPN* expression and clinical features of GC patients with respect to tumor stage was analyzed using TCGA data on stomach adenocarcinoma (STAD). The results showed up regulation of *ASPN* transcript in stages 1 and 2 combined compared to adjacent normal tissue. Furthermore, patients with stage 3 and stage 4 tumor showed significantly higher *ASPN* expression than patients with stage 1 tumor (p<0.001 and p<0.001, respectively; Fig 3E). However, *DCN* gene expression showed overall downregulation in the TCGA data (Fig 3F). Overall, in the TCGA dataset of STAD, consistent upregulation of *ASPN* expression was statistically associated with increasing tumor stage. ASPN is highly expressed in patient tumor tissues compared to normal tissues, whereas *DCN* showed opposite pattern of gene expression (Fig 3G and 3H).

**Fig 4:**
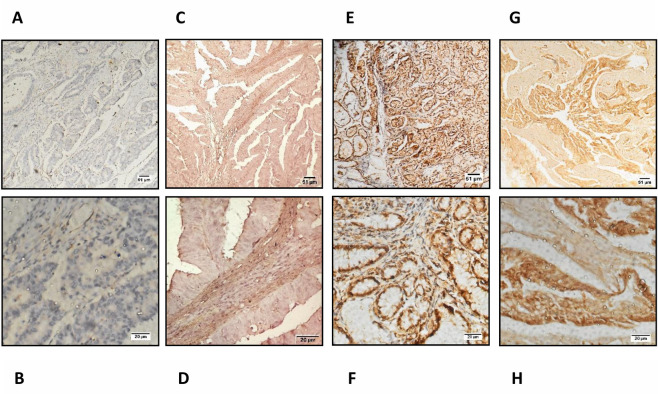
Upregulation of ASPN in gastric tumors: Immunohistochemical staining of ASPN expression in gastric cancer tissue and the criteria for immunohistochemistry scoring. Representative IHC staining comparing ASPN expression in FFPE gastric tissues from GC patients. IHC staining is scored according to semi-quantitative immune-reactivity scoring. Score 0: no staining, score 1: weak staining, score 2: moderate staining, score 3: strong staining. Images of the gastric samples were taken at 100X and 400X magnification In, (A, B) (patient ID# OT63 Normal) Staining Intensity is 0; (C, D) (patient ID# CMCOT02 Tumor) staining intensity is 1; (E, F) (patient ID# 18S292 Tumor) represents the staining intensity of 2 and Fig. (G, H) (patient ID# OT 15 Tumor) represents the staining intensity of 3. Scale bars: 100X = 51μm and 400X = 20 μm. (S4 Table).

To confirm these data at the protein level, we used immunohistochemistry to examine the expression of ASPN in a series of GC samples comprising 12 cases of GC tissues and 4 adjacent normal tissues. Adjacent normal epithelium showed weak or no staining (N = 4), whilst the majority of tumor epithelium showed increased ASPN expression (N = 8; Fig 4).

### Association of *ASPN* expression with clinico-pathological parameters of GC patients

All the 42 patients of GC OT cohort comprised of 31 males and 11 females, ranging in age from 25 years to 80 years, with a mean age of 54 years (Table 1). There were 35 patients of the ENDO cohort, including 28 males and 07 females, ranging in age from 36 years to 85 years, with a mean age of 58 years (S3 Table). Next, the association of *ASPN* expression with clinico-pathological parameters, including patients’ age, gender, tumor size, lymph node metastasis, histological grade, depth of invasion, and clinical stage was examined. All the variables were categorized in Table 1 and statistical significance was evaluated using Fisher’s exact test. The expression of *ASPN* was positively correlated with tumor stage (p = 0.047) and lymph node metastasis (p = 0.008). However, there was no significant correlation between *ASPN* expression and patients’ age or gender (p>0.05).

### Protein-protein interaction predicted by bioinformatics analysis

**Fig 5:**
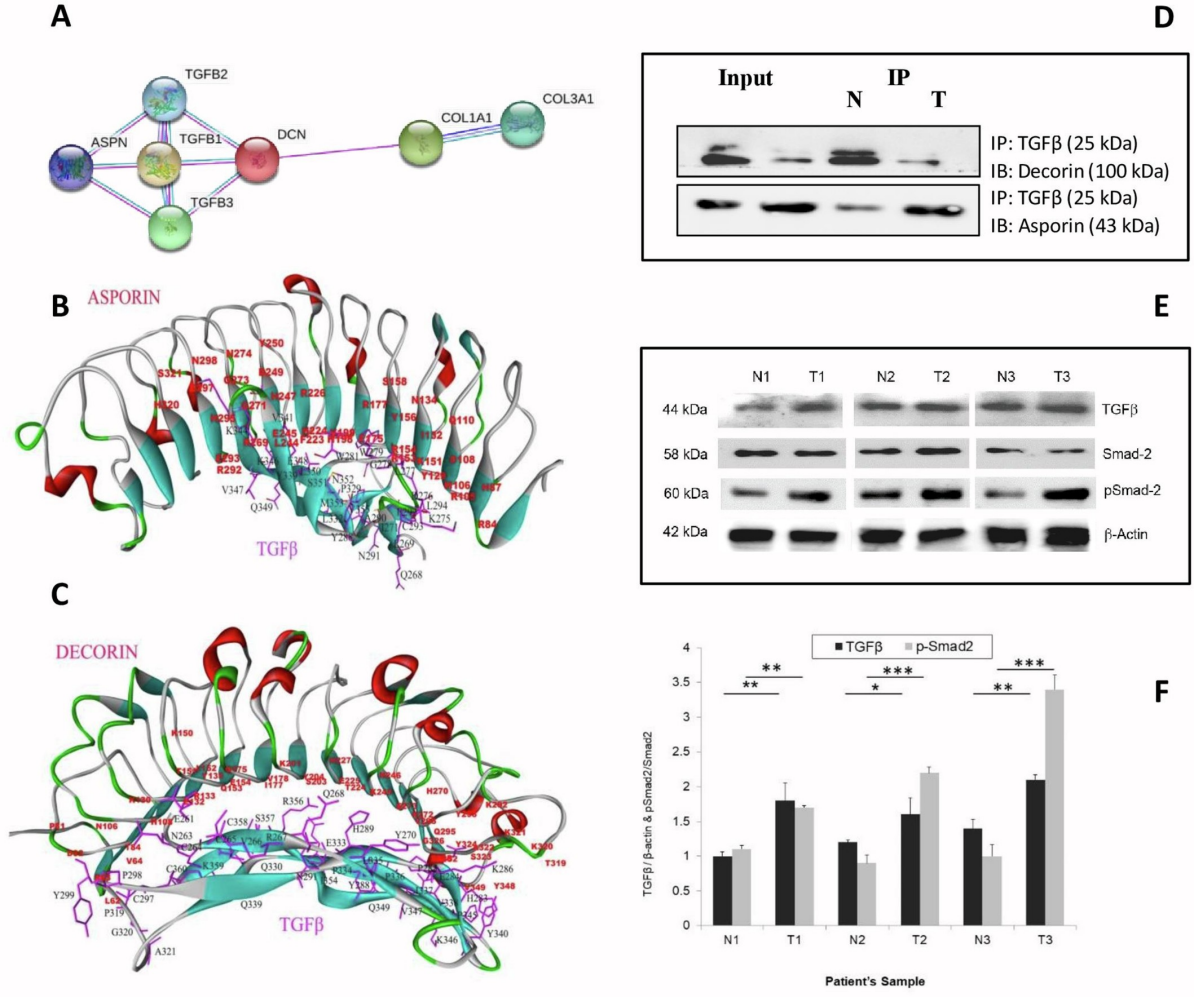
*In silico* protein-protein interaction analysis. (A) String Interaction Analysis *In silico* interaction analysis by String (https://string-db.org/) suggests putative interaction of ASPN and DCN with TGFβ. The protein is represented by a node, and the interaction between paired proteins is represented by an undirected line. (B, C) Patchdock Interaction Analysis: Interaction between TGFβ with ASPN (B) and DCN (C) The interacting residues were shown in figures for ASPN-TGFβ complex and DCN-TGFβ complex respectively, depicting proper binding pose and predicted amino acids were present in the interface area. Amino acid residues from TGFβ are in black font, ASPN and DCN residues are in red font. (D) Co-immunoprecipitation of ASPN and DCN with TGFβ: The co-IP assay revealed an association between ASPN, DCN with TGFβ in gastric tumor and adjacent normal tissues. (E, F) Expression of TGFβ, SMAD2, p-SMAD2 in Gastric adjacent normal and tumor samples analyzed using western blot assay. T1, T2, T3 are tumor tissues and N1, N2, and N3 are respective corresponding normal tissues. Data were quantified, normalized and plotted (n = 3, mean ± SD, *p ≤ 0.05, **p ≤ 0.01, ***p ≤ 0.001, student’s *t-*test).

*In silico* interaction analysis by String (https://string-db.org/) revealed a putative interaction of ASPN and DCN with TGFβ as well (Fig 5A). The Basic Local Alignment Search Tool (BLAST) result of human *ASPN* showed 59.02% sequence identity, 80% query coverage, and E value of 3e-137 with chain A of Biglycan protein from *Bos taurus* (PDB ID: 2FT3). Likewise, human *DCN* showed 90.03% identity in 90% query coverage with A chain of *Bos taurus* DCN protein (PDB ID: 1XKU). ASPN and DCN models obtained from the SWISS model and MODBASE servers showed 0.29Å and 0.22Å insignificant deviations of backbone atoms, respectively. The deviations, however, were quite significant (1.48Å) between ASPN and DCN 3D models of both servers. The stereochemical qualities of both models showed acceptable values in Procheck. In the Ramachandran plot, there were no residues in the disallowed regions. The 95.41% of the residues have averaged 3D-1D score > = 0.2 in the DCN model whereas the Verify3D score for ASPN was 95.03%. The overall ERRAT score for ASPN and DCN were 73.1 and 88.2, respectively. After docking with TGFβ in three various algorithms, all the complexes were analyzed for proper docking pose in terms of interacting residues, surface areas, H-bonds, lowest binding free energy (ΔG) and dissociation constant (Kd). The PatchDock docked complexes of both ASPN and DCN with TGFβ were found to be most acceptable in comparison to others. In comparison to the ASPN-TGFβ complex, it was observed that the DCN-TGFβ complex showed maximum interface area, greater solvent-accessible interface area, higher number of H-bonds, and lower binding free energy and dissociation constant (S2 Table). The interacting residues are shown in Fig 5B and 5C for ASPN-TGFβ complex and DCN-TGFβ complex, respectively, depicting proper binding pose and predicted amino acids present in the interface area.

### TGFβ interacts with ASPN in gastric tumor tissues and DCN in normal gastric tissues

To investigate how ASPN promotes GC progression, a proteomic approach was implemented with the ASPN/DCN-TGFβ complex that was pulled down using an antibody against TGFβ. ASPN and DCN protein levels were measured in patient-derived GC tissues and adjacent normal tissues. Co-IP assays were performed to assess whether ASPN and DCN could physically interact with TGFβ in GC tumor and normal tissue. The results indicated that DCN and TGFβ formed a complex and possibly interacted with each other in normal tissues, whereas ASPN and TGFβ predominantly interacted with each other in gastric tumor tissues (Fig 5D).

### ASPN possibly activates canonical TGFβ signaling pathway

To comprehend the function of ASPN in the TGFβ signaling pathway, the effects on tumor and normal tissues were determined in 3 tumor/normal sample pairs. A total of 30 μg of protein was analyzed using antibodies specific for SMAD2 or phosphorylated SMAD2 (p-SMAD2). Results indicated that p-SMAD2 was significantly enhanced in patient tumor tissues compared to normal tissues, indicating probable activation of the canonical TGFβ pathway (Fig 5E and 5F).

### Increased *ASPN* expression positively correlates with expression of EMT markers

**Fig 6:**
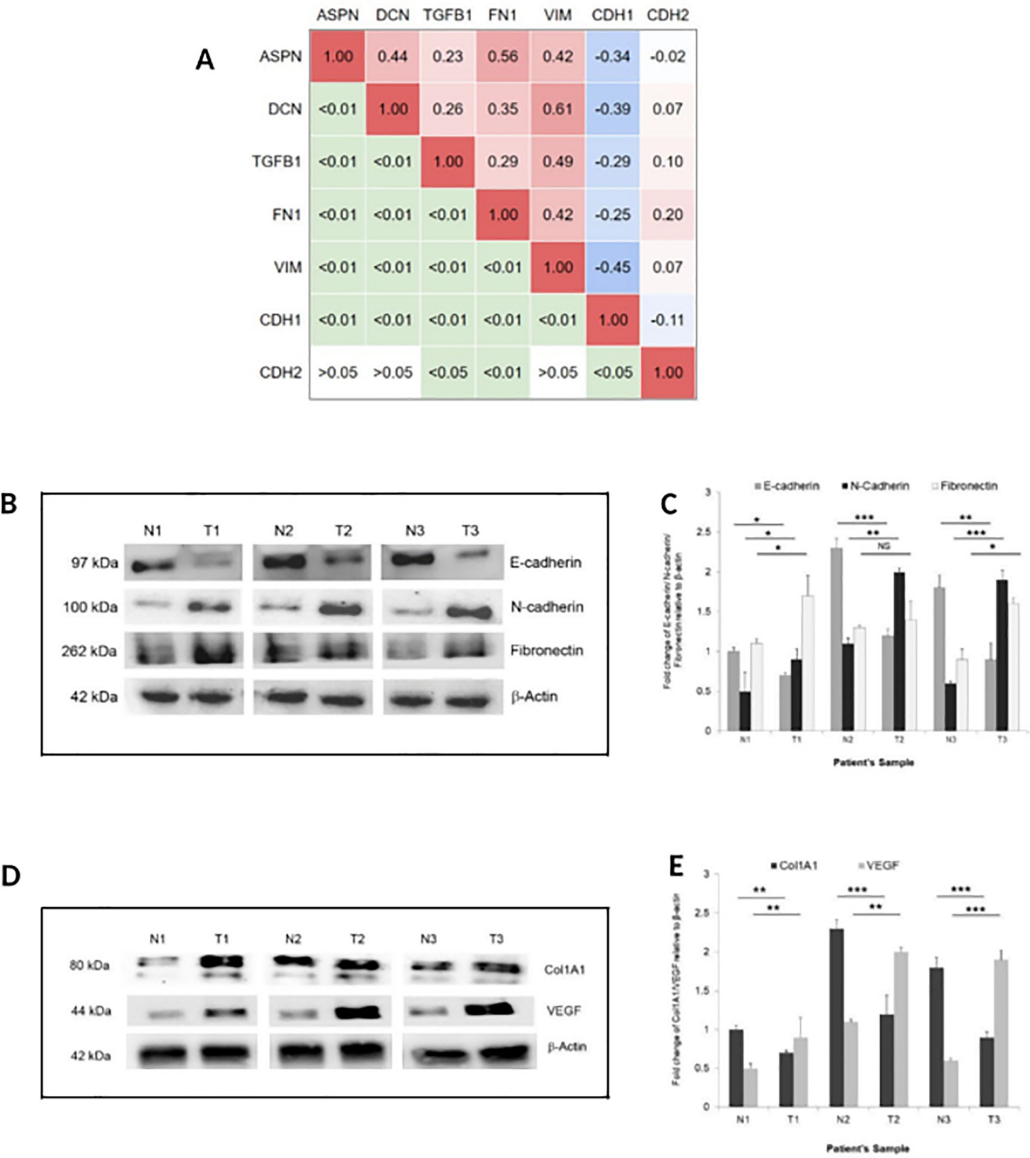
Correlation between the SLRPs and EMT. (A) Correlation Matrix of ASPN, DCN, TGFβ, and several EMT genes on TCGA-STAD data. The right half of the matrix represents Pearson’s correlation coefficient values. The left half of the matrix represents P-values. (B, C) Western blot analysis of E-cadherin, N-cadherin, and fibronectin. Data were quantified, normalized with β-actin and plotted (n = 3, mean ± SD, *p ≤ 0.05, **p ≤ 0.01, ***p ≤ 0.001, student’s *t-*test). (D, E) Expression of angiogenesis marker Collagen (Col1A) and vascular endothelial growth factor (VEGF) in gastric tumor and adjacent normal tissue using western blot assay. T1, T2, T3 are tumor tissues and N1, N2, and N3 are respective corresponding normal tissues. Data were quantified, normalized with β-actin and plotted (n = 3, mean ± SD, *p ≤ 0.05, **p ≤ 0.01, ***p ≤ 0.001, student’s *t-*test).

Correlation of ASPN, DCN, TGFβ, and several EMT genes were derived from TCGA-STAD data (Fig 6A). We hypothesized that enhanced expression of *ASPN* in GC potentially affects cancer cell migration through activation of EMT. Hallmarks of EMT include loss of cell-cell adhesion, reorganization of the actin cytoskeleton, diminished expression of E-cadherin and enhanced expression of mesenchymal markers, such as N-cadherin and fibronectin. As expected, GC tumor tissues showed decreased expression of E-cadherin along with increased expression of N-cadherin and fibronectin (Fig 6B and 6C).

### Enhanced ASPN expression correlates positively with VEGF expression

Given our observation that increased expression of ASPN in gastric tumor tissue triggers EMT phenotype and copiously produces collagen I (Fig 6D), distinct expression changes associated with angiogenesis were determined by evaluating the expression of VEGF. Results suggested that ASPN expression is positively correlated with the protein level of VEGF (Fig 6E).

## Discussion

ASPN is a rather newly identified extracellular matrix (ECM) protein that acts as oncogenic protein in most solid tumors [21, 47]. However, in triple negative breast cancer, it emerged as a tumor suppressor [18], defining its tissue-specific functional variability. In GC cell lines, it has been shown that ASPN is significantly upregulated and promotes proliferation of GC cells by interacting with the Proteasome 26S Subunit, Non-ATPase 2 (PSMD2) [48]. Recently, abundant evidence has affirmed the crucial role of ASPN in EMT in multiple cancers [47, 49].

ASPN is predominantly secreted from cancer-associated fibroblasts (CAFs) in a variety of tumors and is correlated with tumor invasion and metastasis [50]. Satoyoshi et al. (2015) showed that ASPN secreted from CAFs have essential roles in co-ordinated invasion in scirrhous gastric carcinoma, which causes wall thickening of the stomach by spreading from the lining to the muscle of stomach [51]. Unlike breast cancer, where ASPN was found to play dual opposing roles, in GC patients high tumor-specific expression of ASPN exhibited worse clinico-pathological parameters and survival rates [52–54]. Analysing TCGA and GEO datasets of GC patients, our study shows that GC patients with high *ASPN* levels had a worse 5-year overall survival (OS) compared to patients with low *ASPN* levels.

Herein, we systematically examined the functional role of ASPN in GC tissue in our cohort. Our, findings are in accordance with previous studies. The TCGA-STAD cohort, revealed a significantly elevated *ASPN* gene expression in GC tissues vs matched adjacent non-tumor tissues, while reduced expression of DCN was found in cancer tissues vs normal non-tumor tissues. Despite sharing almost 60% identical amino acid sequence [55], ASPN and DCN have been shown to express antagonistically in GC.

Interactions between ASPN and TGFβ have been subjected to intense investigations in various bone and cartilage diseases; however, the mechanism of action is vastly different in GC [56–58]. It has been shown in bone and ligament diseases that ASPN inhibits the TGFβ ligand (TGFB1) [30, 58]. Our study, however, demonstrated for the first time that the association of TGFβ is higher with ASPN compared to that with DCN in GC tissue, as demonstrated by the co-immunoprecipitation assay. Since ASPN regulates GC metastasis through activation of EGFR and the ERK-CD44/MMP-2 pathway [47], it perhaps favourably associates with TGFB1 and activates the signaling pathway to promote tumor growth and progression. Interestingly, based on in *silico* protein-protein interaction analyses, various parameters, interacting residues, greater solvent-accessible interface area, lowest binding free energy (ΔG), and dissociation constant (Kd), revealed that the DCN-TGFβ complex showed better binding in comparison to ASPN-TGFβ complex. Reduced expression of DCN in GC tissues reduced availability for interaction with TGFβ and hence, diminished the involvement of DCN in the TGFβ signaling pathway, further endorsing the tumor suppressive role of DCN in GC.

Our results thereby indicate that over expression of ASPN in GC tumor tissue and its enhanced association with TGFβ probably triggers the canonical TGFβ signaling pathway, which was reflected by significantly higher levels of phosphorylated pSMAD2 compared to adjacent normal tissue. This intriguing and novel cross talk between SLRPs and TGFβ deserves further investigation in several GC cell lines and patient tissues.

Overexpression of ASPN has been found demonstrated in later stages of GC and we hypothesized that ASPN is perhaps a significant contributor to the metastatic phenotype. A recent study on prostate cancer by Hurley et al. (2016) on *ASPN*-null mice delineated that *ASPN* impacts both tumor and the tumor microenvironment, promoting metastasis [59]. In our study, we showed that over expression of ASPN in tumor tissue is correlated with high expression of mesenchymal proteins such as N-cadherin, fibronectin, and low expression of the epithelial marker protein—E-cadherin, indicates metastasis in tumor tissues.

Previous studies have shown Collagen type I can reduce cell-to-cell adhesion and enhance migration by inducing disassembly of the E-cadherin/catenin complex in gastric carcinoma cells [60]. Here we found that Collagen I is markedly increased in GC tissue and correlates with high ASPN expression. VEGF, the major angiogenic growth factor, and its association with GC were also assessed, since angiogenesis mostly accompanies aggressiveness and metastasis of GC. Our results have significantly established a positive correlation between over expression of ASPN and VEGF in GC tissues. Therefore, enhanced expression of ASPN, along with both Collagen I and VEGF, strongly indicates increased metastasis and disease progression.

**Fig 7:**
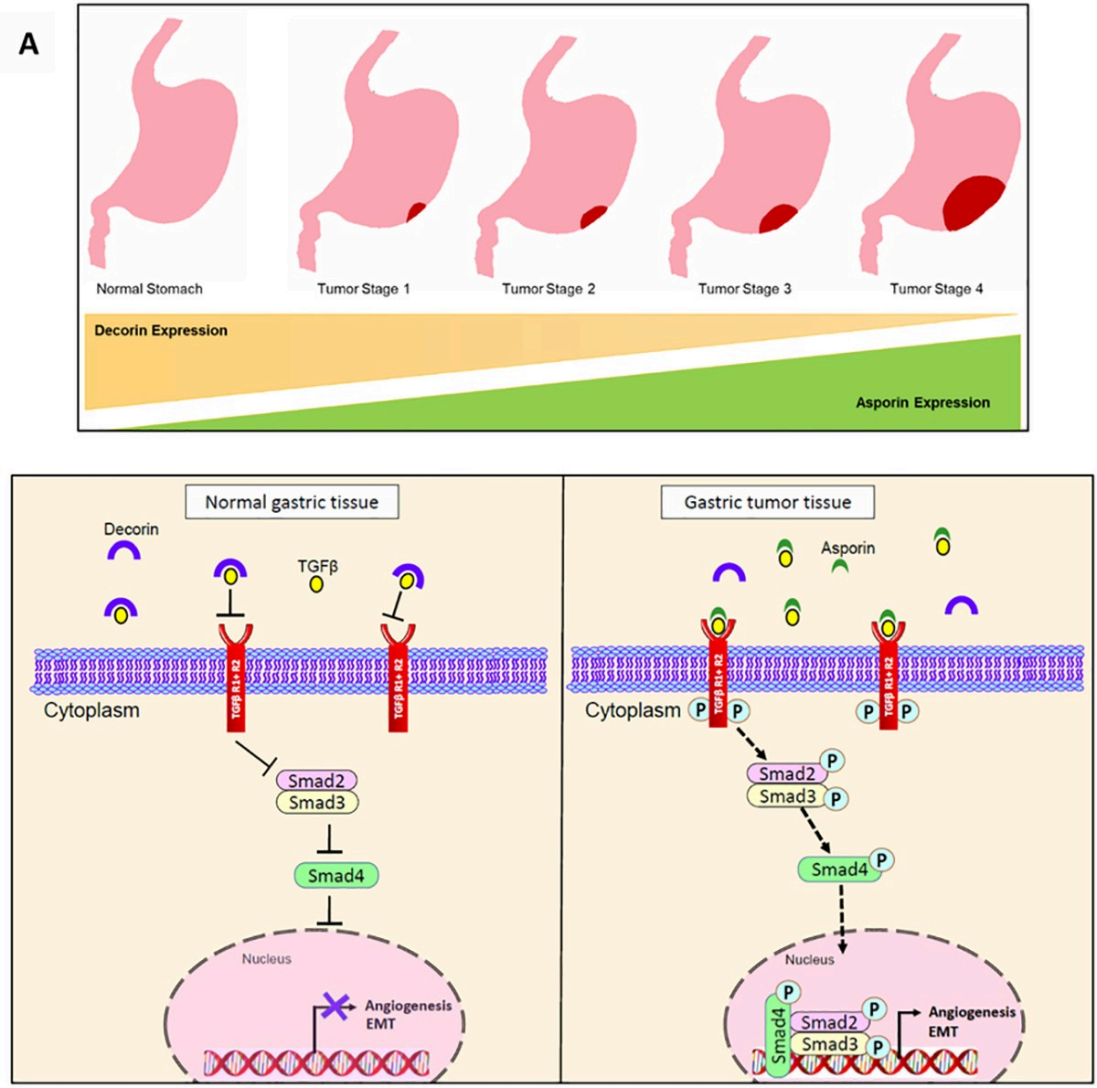
Model for regulatory mechanism of ASPN and DCN: We have proposed a Schematic illustration of the mechanism based on our conclusion. A. In normal gastric epithelium, ASPN is not present in the tumor microenvironment, DCN exerts its inhibitor activity by binding with TGFβ ligand, thereby, impede TGFβ signaling cascade and subsequent EMT, angiogenesis. (B). In GC tissue, DCN expression is lacking, ASPN binds with TGFβ ligand, triggers the canonical pathway by phosphorylating Smad2 and Smad3. Phosphorylated Smads translocate to the nucleus and activates EMT and angiogenesis. Blunted lines, inhibition; dotted line, activation; cross, disrupted interaction.

Our study is the first to indicate a putative complex interaction between ASPN, DCN and TGFβ in the context of GC development. ASPN is highly over expressed in majority of GC cases and associates with TGFβ may facilitate the activation of the canonical TGFβ pathway, which is otherwise suppressed in normal gastric epithelium. On the other hand, DCN-bound TGFβ presumably prevents pathway activation and thereby acts as a tumor suppressor. Increased expression of the canonical TGFβ pathway component pSMAD2 is also correlated with up regulation of mesenchymal genes, collagen fibrillogenesis, and angiogenesis, all of which triggers enhanced metastasis in GC tissues, as summarized in Fig 7. Our data therefore justifies the use of SLRPs as prognostic biomarkers in GC and expand its possible clinical importance. However, knockdown studies of asporin and decorin in GC cell lines and evaluation of their effect on TGFβ deregulation will be helpful to understand the biological contributions of asporin and decorin in of GC.

## Conclusions

In conclusion, the present study is the first to investigate roles of asporin, decorin and their interaction with TGFβ in gastric cancer tissue and corresponding normal tissues. Our findings indicated that overexpressed *ASPN* interacts with TGFβ in gastric tumor epithelium and possibly triggers the EMT. However, it is necessary to conduct further studies in larger gastric cancer cohorts and in gastric cancer cell lines to confirm our results. Our study provides a basic reference for ASPN, DCN and TGFβ in GC.

## Supporting information

S1 Fig Expression of DCN: Gene expression analysis using quantitative real-time PCR suggests DCN is under expressed in gastric tumor tissue compare to adjacent normal in our OT cohort. (DOCX)

S1 File Materials and methods: Bioinformatics prediction and modeling of protein-protein interaction. (DOCX)

S1 Table Primer sequence table. (XLSX)

S2 Table The interaction comparison of TGFβ protein with ASPN and DCN. (DOCX)

S3 Table Endo cohort patient’s data. (XLSX)

S4 Table IHC patient’s details. (XLSX)

S5 Table Expression fold changes of 58 genes with respect to adjacent normal samples that showed stage-wise upregulated in TCGA-STAD. (XLSX)

S1 Raw images (PDF)

## Abbreviations

TGFβ: Tumor Growth Factor Beta
ASPN: Asporin
CAF: Cancer-associated Fibroblasts
DCN: Decorin
ECM: Extracellular Matrix Protein
EMT: Epithelial Mesenchymal Transition
GEO: Gene Expression Omnibus
SLRP: Small Leucine Rich Proteoglycan
TCGA-STAD: The Cancer Genome Atlas Stomach Adenocarcinoma
VEGF: Vascular Endothelial Growth Factor
