# Supplementary material for: Reciprocal interplay between asporin and decorin: Implications in gastric cancer prognosis
Source: PLoS One. 2021 Aug 11;16(8):e0255915. doi: 10.1371/journal.pone.0255915 (PMC8357146; doi:10.1371/journal.pone.0255915)

**S1 Fig. Expression of *DCN*: Gene expression analysis using quantitative real-time PCR suggests *DCN* is under expressed in gastric tumor tissue compare to adjacent normal in our OT cohort.**


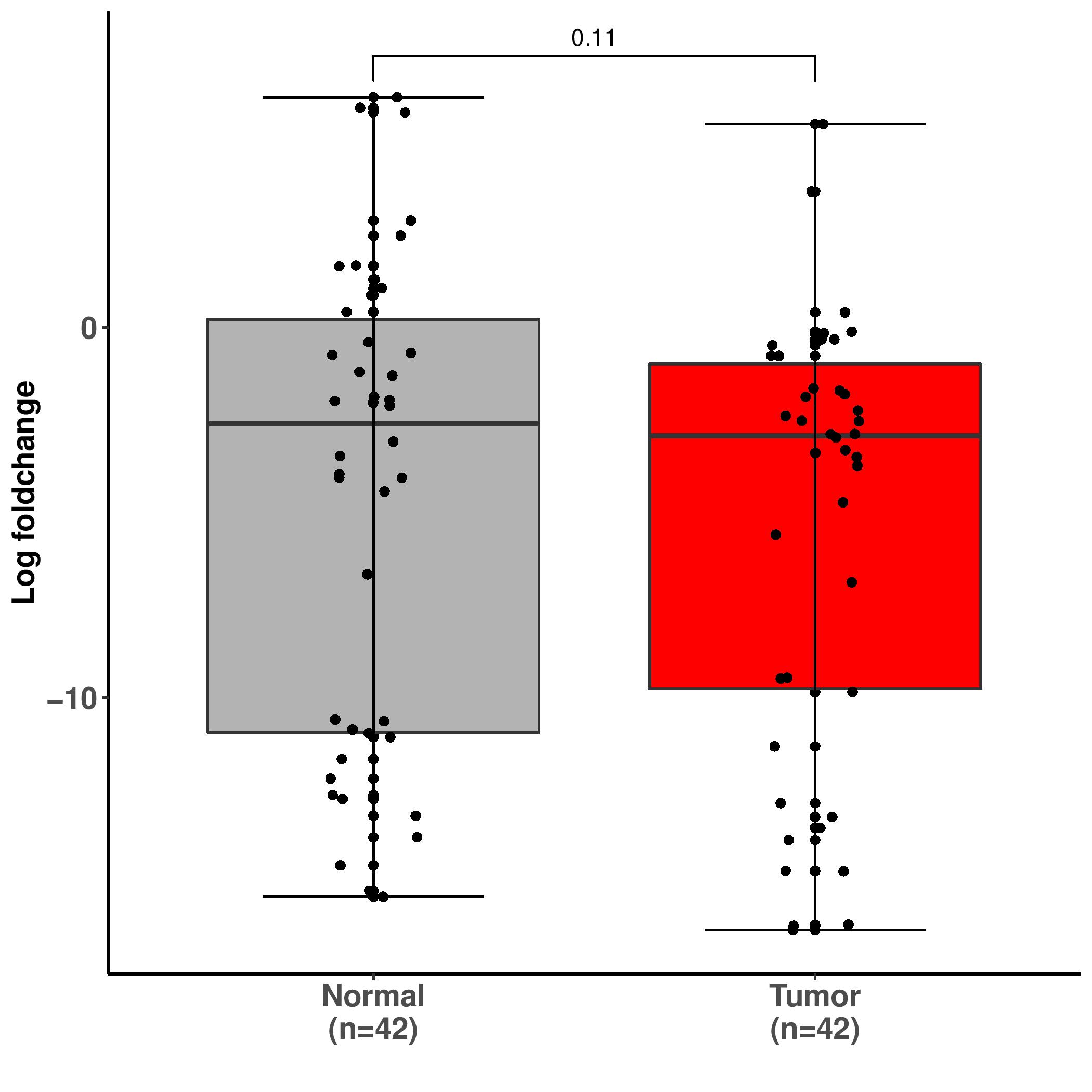

Supplement: S1 Fig — (DOCX) [file pone.0255915.s001.docx]
