# Supplementary material for: Reciprocal interplay between asporin and decorin: Implications in gastric cancer prognosis
Source: PLoS One. 2021 Aug 11;16(8):e0255915. doi: 10.1371/journal.pone.0255915 (PMC8357146; doi:10.1371/journal.pone.0255915)
Supplement: S1 File — (DOCX) [file pone.0255915.s002.docx]

**S1 File: Materials and Methods:** Bioinformatics prediction and modeling of protein-protein interaction (DOCX)
