# Supplementary material for: Reciprocal interplay between asporin and decorin: Implications in gastric cancer prognosis
Source: PLoS One. 2021 Aug 11;16(8):e0255915. doi: 10.1371/journal.pone.0255915 (PMC8357146; doi:10.1371/journal.pone.0255915)
Supplement: S2 Table — (DOCX) [file pone.0255915.s004.docx]

**S2 Table. The interaction comparison of TGF**β **protein with ASPN and DCN**

| **Interaction Summary** | **ASPORIN** | **DECORIN** |
| --- | --- | --- |
| **Interface Area (Å^2^)** | 1359.6 | 1549.1 |
| **No. of H-Bonds** | 11 | 15 |
| **No. of Salt bridges** | 5 | 2 |
| **No. of interface**  **residues (in %)** | 14.9 (ASPN)  45.1 (TGFβ) | 14.8 (DCN)  52.4 (TGFβ) |
| **Solvent accessible interface area (in %)** | 8.8 (ASPN)  22.7 (TGFβ) | 11.1 (DCN)  25.7 (TGFβ) |
| **H-Bonds**  **(Acceptor: Donor)** | 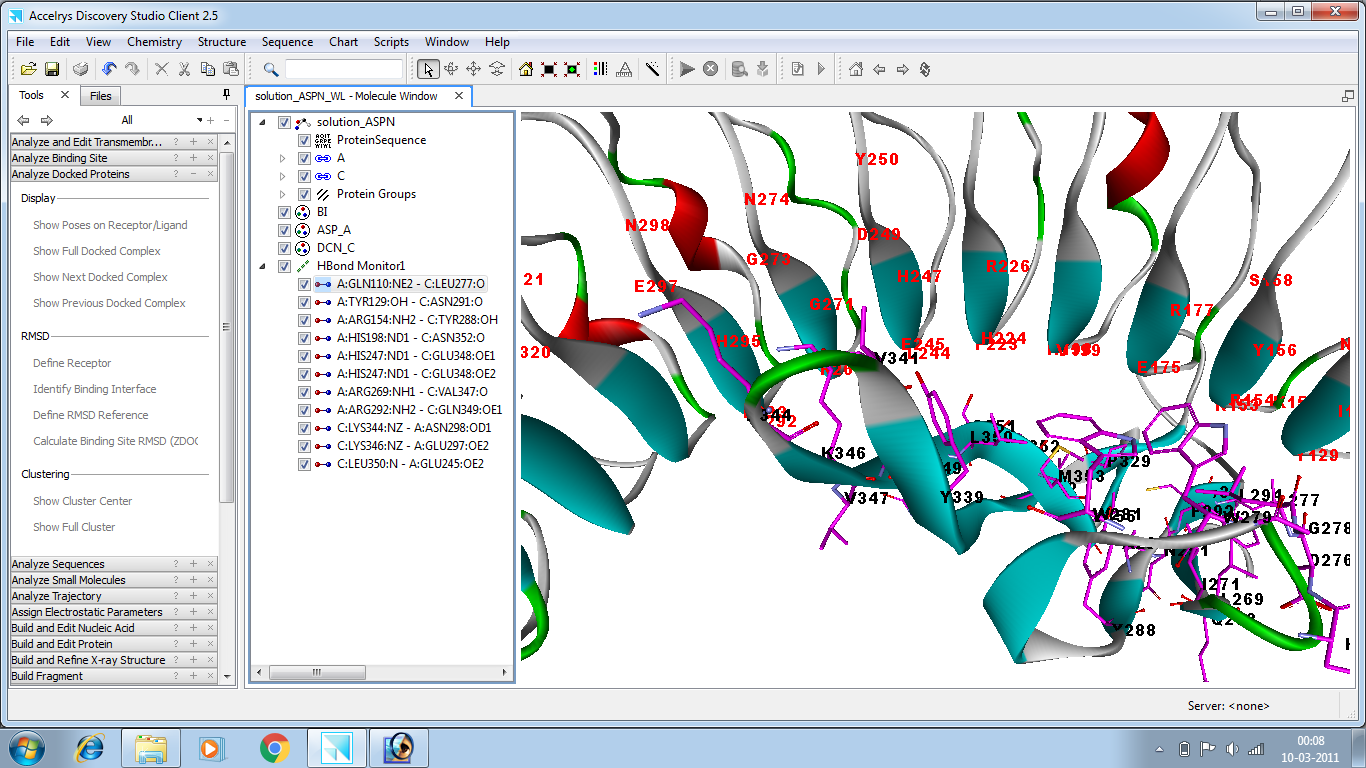 | 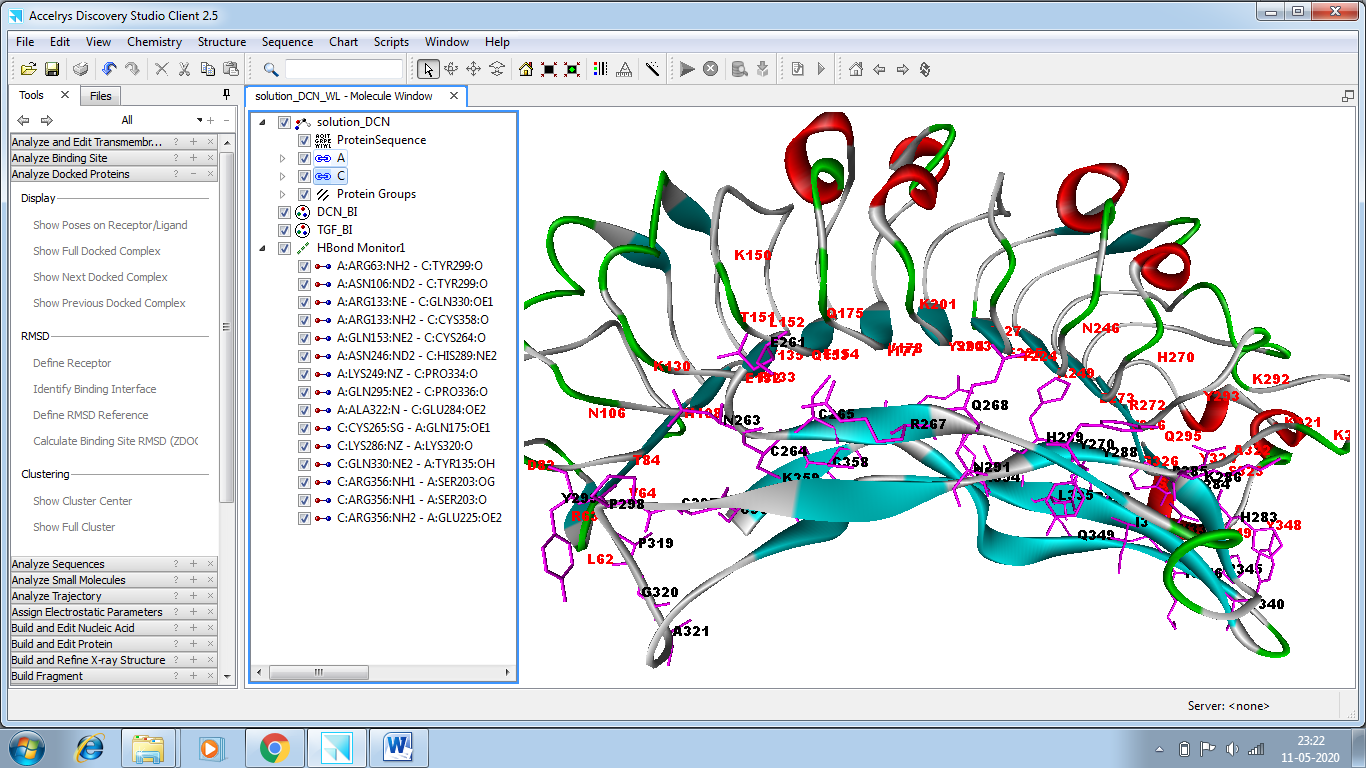 |
| **Hydrophobic Interactions within 5Å** | 106 MET A: 269 LEU C  129 TYR A: 269 LEU C  129 TYR A: 271 ILE C  129 TYR A: 292 PHE C  132 ILE A: 277 LEU C  156 TYR A :277 LEU C  156 TYR A: 279 TRP C  199 VAL A: 350 LEU C  199 VAL A: 353 MET C  223 PHE A: 350 LEU C  223 PHE A :353 MET C  250 TYR A: 341 VAL C | 177 ILE A: 266 VAL C  293 TYR A: 285 PRO C  296 VAL A: 336 PRO C  348 TYR A: 340 TYR C  348 TYR A: 345 PRO C  349 VAL A: 345 PRO C  349 VAL A: 347 VAL C  352 ALA A: 347 VAL C |
| **ΔG (kcal mol^-1^)** | -14.5 | -19.0 |
| **K_d_ (M) at 25.0 ℃** | 2.2E-11 | 1.1E-14 |
