# Supplementary material for: Reciprocal interplay between asporin and decorin: Implications in gastric cancer prognosis
Source: PLoS One. 2021 Aug 11;16(8):e0255915. doi: 10.1371/journal.pone.0255915 (PMC8357146; doi:10.1371/journal.pone.0255915)
Supplement: S1 Raw images — (PDF) [file pone.0255915.s008.pdf]

Fig 3G

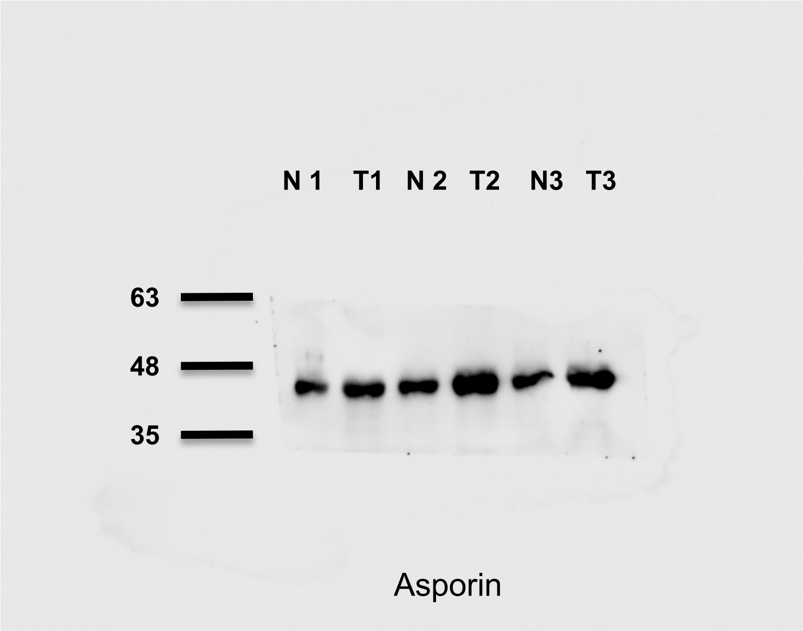

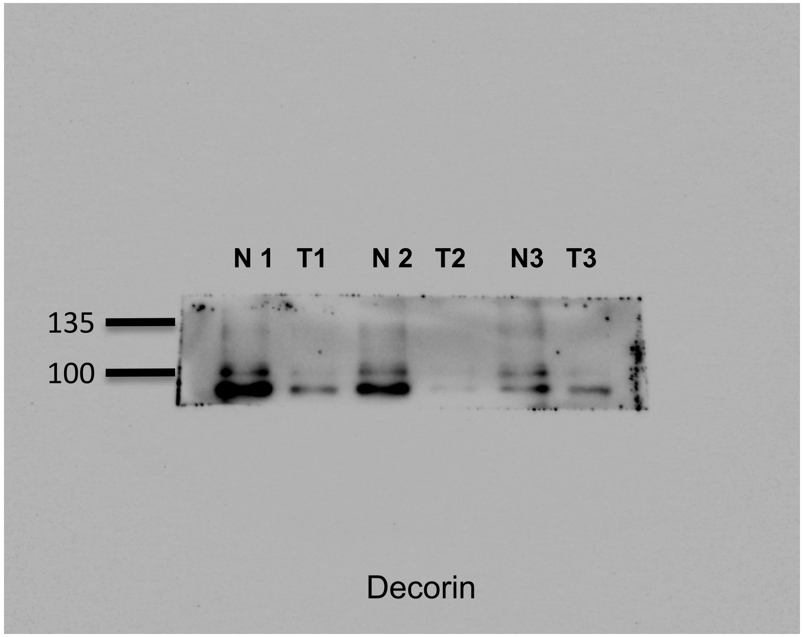

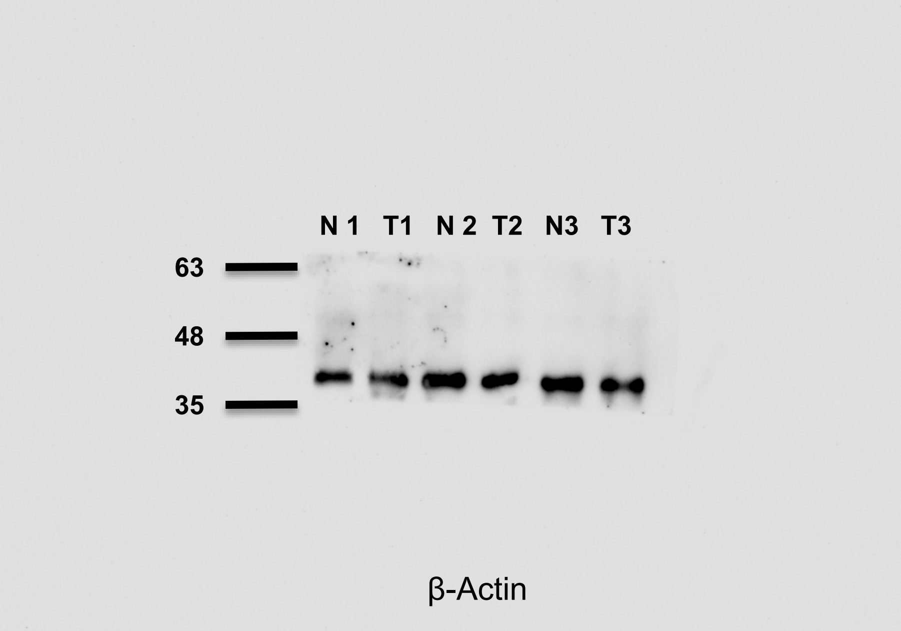

All bands were detected using chemiluminescence

Fig 5D

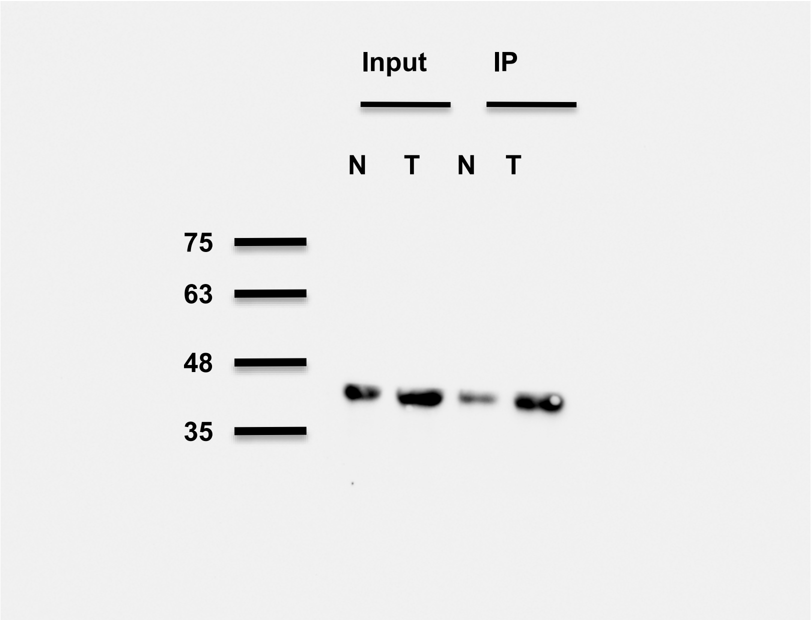

IB: Asporin IP: TGFβ

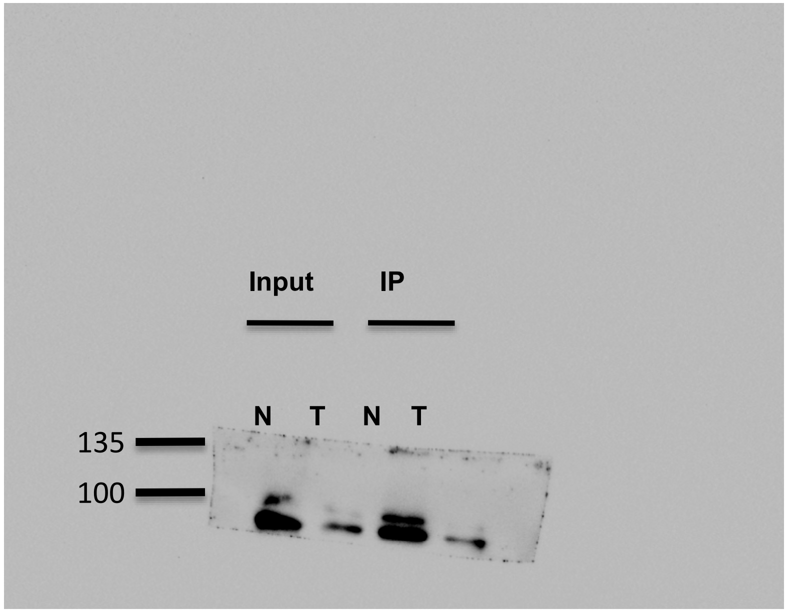

IB: Decorin IP: TGFβ

All bands were detected using chemiluminescence

Fig 5E

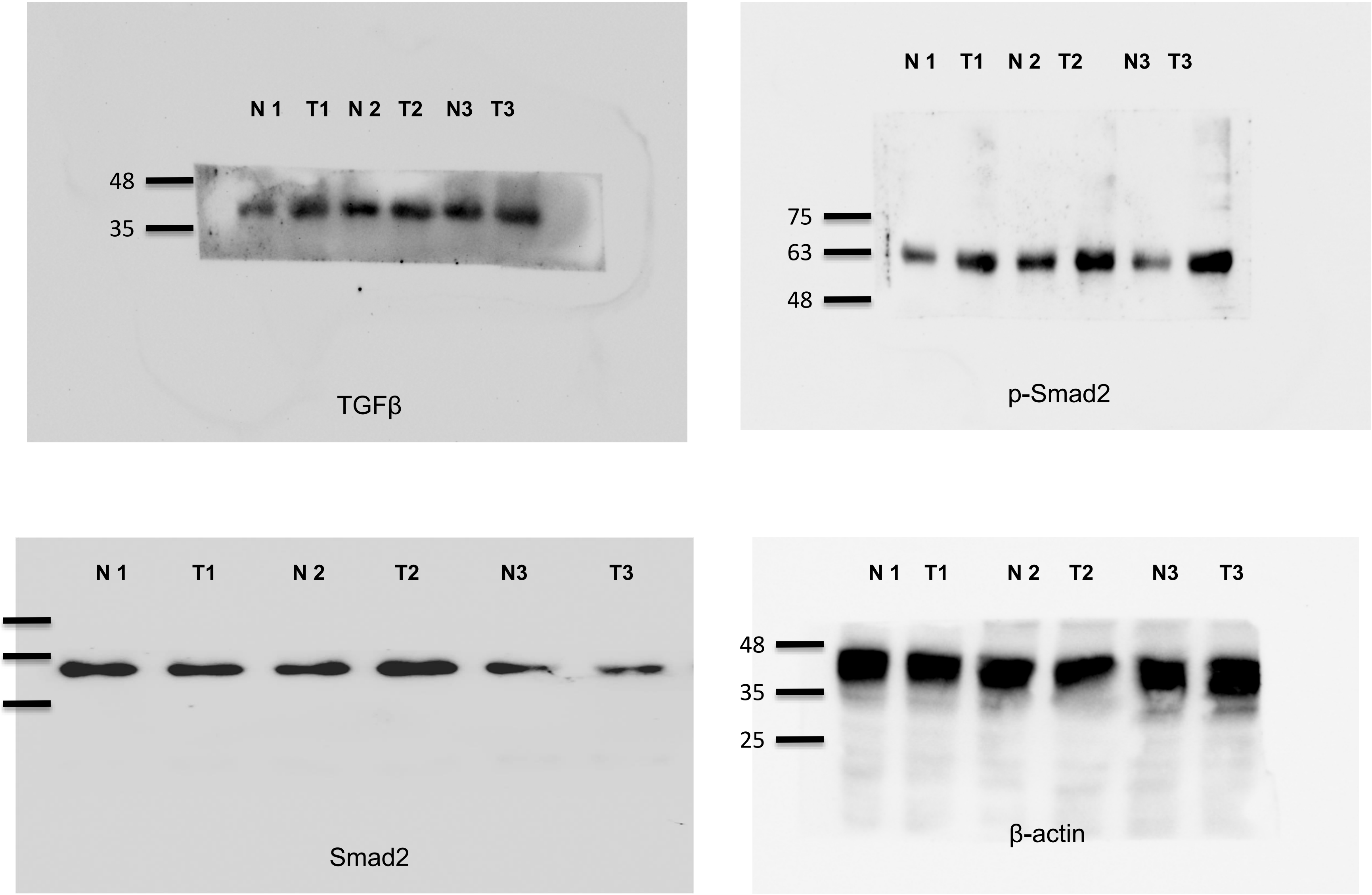

All bands were detected using chemiluminescence

Fig 6B

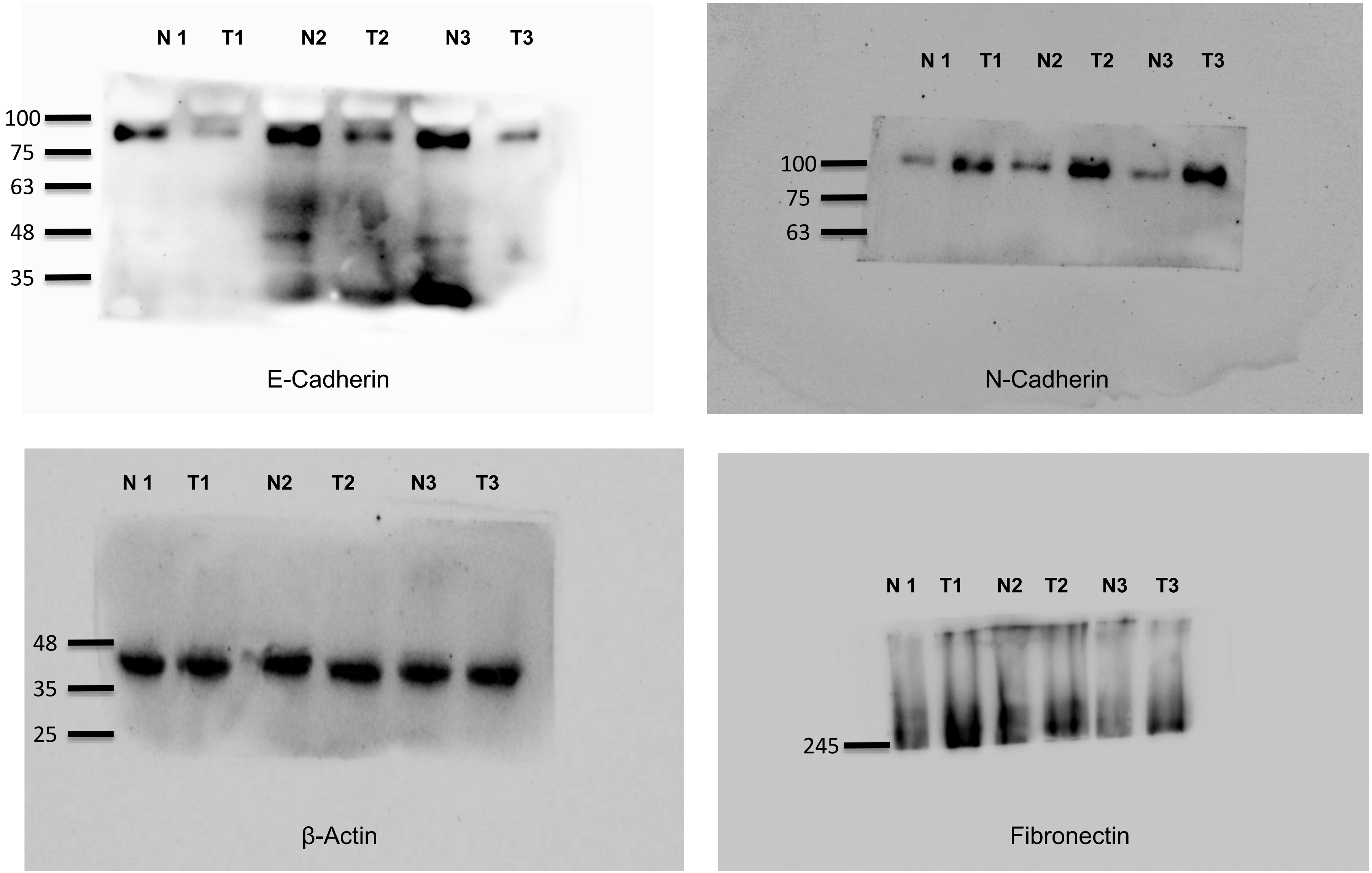

All bands were detected using chemiluminescence

Fig 6D

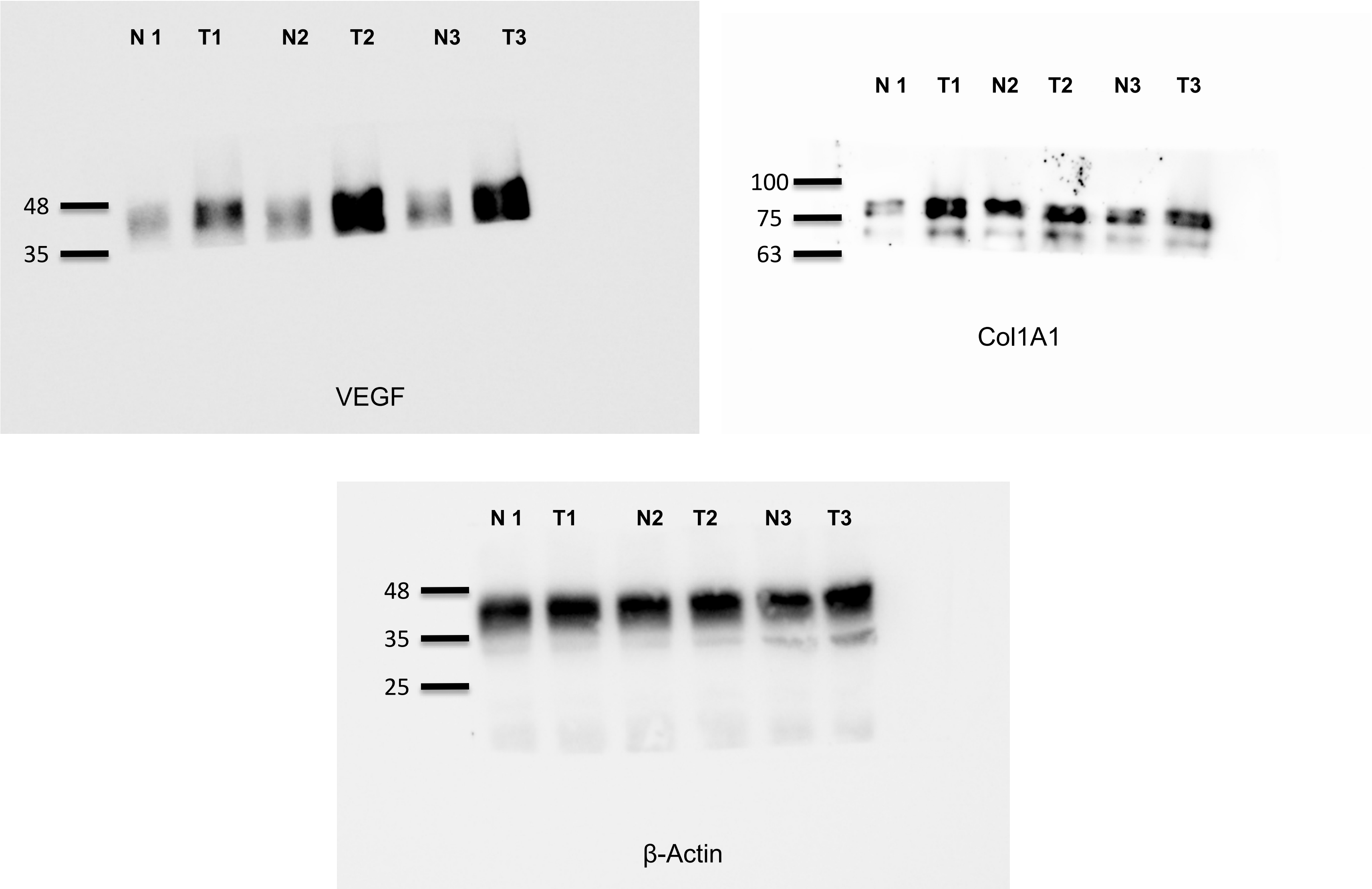

All bands were detected using chemiluminescence
